# Psychological care needs for frontline nurses during the COVID-19 pandemic: A qualitative study

**DOI:** 10.3389/fpubh.2022.1043515

**Published:** 2022-11-10

**Authors:** Chuanqi Ding, Limin Wang, Zhiting Guo, Yun Chen, Jingfen Jin

**Affiliations:** ^1^Department of Emergency, Changxing County People's Hospital, Huzhou, China; ^2^Department of Nursing, Tongji Medical College, Union Hospital, Huazhong University of Science and Technology, Wuhan, China; ^3^Department of Nursing, The Second Affiliated Hospital Zhejiang University School of Medicine, Hangzhou, China; ^4^Department of Public Health, Changxing County People's Hospital, Huzhou, China; ^5^Key Laboratory of the Diagnosis and Treatment of Severe Trauma and Burn of Zhejiang Province, Hangzhou, China

**Keywords:** COVID-19, nurses, psychological care, needs, qualitative research

## Abstract

**Background:**

In the course of the COVID-19 pandemic, nurses have played vital roles in clinical treatment. Their success in providing adequate care services depends on their psychological state, which determines their physical health, work status, therapeutic outcomes, and response to public health emergencies. However, a limited number of studies have evaluated psychological care needs from the perspective of nurses. This study aimed to describe the psychological care needs for frontline nurses in the course of the COVID-19 pandemic.

**Methods:**

This was a qualitative descriptive study. Data were collected through semi-structured in-depth interviews with 15 frontline nurses who had been involved in the care of COVID-19 positive patients during the COVID-19 pandemic, and received psychological care. The conventional content analysis was used to identify themes from the interview transcripts.

**Results:**

Four major themes about the psychological care needs of frontline nurses were identified: (1) psychological service providers (categories: professional service team, trustworthy person or group, ability to empathize with nurses); (2) problems with psychological care (categories: lack of universal screening and focused attention, online group counseling lacks targeting, psychological interventions lack individualization); (3) psychological care content (categories: mental health-related education, recognition of nurses' contributions, problem-solving therapy, psychological counseling and venting); (4) organization and management of psychological services (categories: focus on the psychological care needs of frontline nurses, build a standardized psychological service process system).

**Conclusion:**

It is important to understand individual psychological care needs of frontline nurses and to provide them with tailor-made psychological care that meet their needs. This will improve their mental health, promote clinical care and quality responses to public health emergencies.

## Introduction

The COVID-19 pandemic has exerted major challenges to health care systems around the world, and caused major psychological and physical shocks to people. Nurses have played a vital role in the treatment of COVID-19. However, due to the heavy workload, extreme physical labor, high risk of infection, fear of family transmission, loss of patients and colleagues, as well as long-term isolation from family members and other reasons, they are more likely to exhibit higher degrees of psychological distress (1–4).

In the clinical environment, contact time between nurses and patients is longer, the frequency of physical contact is higher, which increases the possibility of cross-transmission (1). Due to the long exposure time of frontline nurses, they are more likely to exhibit varying degrees of mental disorders (5). Studies had shown that in the early stages of the COVID-19 pandemic, the prevalence of depression, anxiety and sleep disorders among frontline medical staff was 50.4, 44.6, and 34.0%, respectively. Other psychological distress associated symptoms were as high as 71.5% (3).

Previous studies had shown that, in clinical work, nurses bear the greatest psychological pressure (6), experience higher levels of anxiety (7), are more likely to develop post-traumatic stress disorders (PTSD) (8), and exhibit higher incidences of depression (9), with 6.5% of the nurses expressing suicidal ideas (2). These mental health disorders affect the attention, understanding, decision-making ability, work performance as well as long-term overall health of nurses (2, 10, 11). The decline in nurses' mental and physical health conditions seriously impacted on the performance of their duties and negatively affects health care performance (12–14). These factors have a direct effect on the health of patients since nurses with mental health problems may be less involved in patient interactions, and make more medical errors, etc., which may damage the clinical outcomes of patients (15–18).

Since nurses play an important role in preventing and controlling infections as well as curbing public health incidents, maintaining their mental health is of great significance to controlling infectious diseases (10, 19). Therefore, it is important to describe the psychological care needs of nurses. Studies have reported that attention should be paid to the special psychological care needs of frontline nurses, and that they should be given tailor-made psychological care services (20–22). Rajkumar (23) highlight that time-limited and culturally sensitive mental health interventions tailored to frontline nurses should be developed. However, few studies have delved into determining what kind of psychological care frontline nurses need, and whether the psychological services they receive fully meet their expectations. A survey in Wuhan showed that 3,556 (75.8%) nurses believed that it is necessary to regularly participate in individual or group consultations during the outbreak, while 363 (7.7%) nurses said they needed the help of mental health professionals (2). Another study used a questionnaire survey to determine the psychological care content that nurses are most interested in, the psychological resources most anticipated, and who the participants hoped to receive mental care from Kang et al. (10). These studies are limited by the fact that they adopted structured questionnaire surveys, and they did not involve firstline nurses who could have been better placed to use their language and experience to express their individual views on the need for psychological services. Nekooei Moghaddam et al. (24) conducted interviews with 23 disaster relief nurses who had overseen an earthquake rescue mission. These nurses mentioned the need for psychological care. They needed someone to talk to and accompany them, to tell them if they had any psychological problems, to know their existence and to maintain a good mental state.

Previous studies have not specifically explored the psychological care needs for frontline nurses in China or other countries. This study was designed to explore the psychological service needs of nursing staff directly from frontline nurses during the COVID-19 pandemic using qualitative interviews to listen and understand the anxiety and distress experienced by frontline nurses during the COVID-19 pandemic, and to provide suggestions and references for providing individualized psychological services to nurses.

## Materials and methods

### Study design

To better understand the psychological care needs for frontline nurses in the course of the COVID-19 pandemic, this study used a qualitative descriptive approach with a constructionist epistemology to provide comprehensive information about an event (25). This epistemology acknowledges that knowledge is constructed from an individual's perception and experiences, and constructed via speech to understand the world (26). Our study adopted this epistemology as meanings can emerge from the active engagement of the researcher with the participant through a bidirectional understanding of the experience relationship, where language is viewed as implicit in the social production and reproduction of both meaning and experience (27). The study was reported according to the COREQ guidelines (28) for qualitative studies (Supplementary File 1).

### Participants

Purposive sampling and maximum difference sampling strategies were used (29). Differences were reflected in education level, age, gender, marriage, working years, etc. These frontline clinical nurses were selected during the COVID-19 pandemic at a tertiary hospital in Wuhan, China. The inclusion criteria were: (a) practicing nurses with a nurse practitioner qualification certificate; (b) working at fever clinics, isolation wards, or square cabin hospitals, and caring of COVID-19 positive patients during the pandemic; (c) receiving various psychological care services during the pandemic, including psychosocial counseling groups and hospital psychological care team. The psychosocial counseling groups are composed of professional psychologists, including experts with experience in post-disaster psychological crisis intervention and mental health experts. The hospital psychological care team is composed of nurses with psychological counseling qualifications from various departments. The team members perform routine clinical nursing work in each department. When nurses have psychological problems, they seek help from the psychological care team members of their department, referred to as mental health nurses.

### Data collection

Data collection by CD and LW. Before the start of the formal interview, nurses who met the sampling criteria were contacted in advance. We gave them the relevant information regarding the study, established friendly relations, and agreed with them about the time, place, and method of the interview. After conducting semi-structured interviews with 15 participants, the data reached saturation. Among them, five participants chose face-to-face interviews while 10 of them chose telephone interviews. None dropped out of the study. The initial interview outline was formulated after systematically reviewing the literature and consulting experts. The final interview outline was established after performing a pilot-interview with 3 respondents, and they were not included among the 15 participants. The final semi-structured interview guide consisted of six open-ended questions aimed at exploring the psychological care they received during the COVID-19 pandemic and their need for psychological care services. (a) How did you seek for psychological help during the epidemic? Can you give some examples of this? (b) What kind of psychological services did you receive from your hospital during the epidemic? (c) What do you think of these psychological services? Do these psychological services help you get out of stress? (d) What do you hope to do to improve psychological care services in public health emergencies? Do you have some good advice? (e) Do you think there are other ways to provide you with psychological help? Please give examples (f) Apart from that, what else do you need to add about psychological care?

Face-to-face interviews were conducted in a hospital environment that was familiar to nurses, in a quiet and undisturbed nurse's lounge, with only the interviewer and interviewee present. The telephone interview was conducted in a quiet room with a good network communication signal. All interviews lasted 40 to 60 min. During the interviews, a combination of simultaneous recording and note recording were used.

### Data analysis

After each interview, the audio recordings were transcribed into verbatim word by word transcripts within 24 h. Data analysis and data collection were conducted simultaneously. Using the qualitative data analysis software NVivo 12 (QSR International Pty Ltd) performed the conventional content analysis by two researchers. Coders (CD and LW) first read the entire transcripts of each participant's interviews several times to obtain an overall understanding of the study phenomenon. Afterwards, the coders hand coded any narrative data related to the participant's psychological care needs on a line-by-line basis. Then, we abstracted meaning units based on the latent meanings behind them via a coding process and developed categories through the comparison of the codes in terms of similarities and differences. The coding of individual transcripts was discussed between the two coders and repeatedly tweaked the ambiguous code fragments until an agreement was reached. The comparison of the categories and reflection on the latent meaning of the data led to the development of some themes.

Based on the coded data, a theme matrix has been constructed. The quotations for each theme were extracted and used to present the participants' expressions of the theme, to facilitate our understanding of the specific meaning of the theme, and to enrich our findings and make them more socially relevant.

### Ethical considerations

The study was conducted in accordance with the Declaration of Helsinki, and approved by the Institutional Review Board of Union Hospital, Tongji Medical College, Huazhong University of Science and Technology (IRB number: 2021S003). Before the interview, participants were informed of the purpose of the study, after which they were required to provide an oral or written consent. Participants were also informed about the privacy of their contributions and their right to withdraw from the study at any given time. Simultaneously, we code-converted interviewee's name and concealed information that could identify them.

## Results

**Figure 1:**
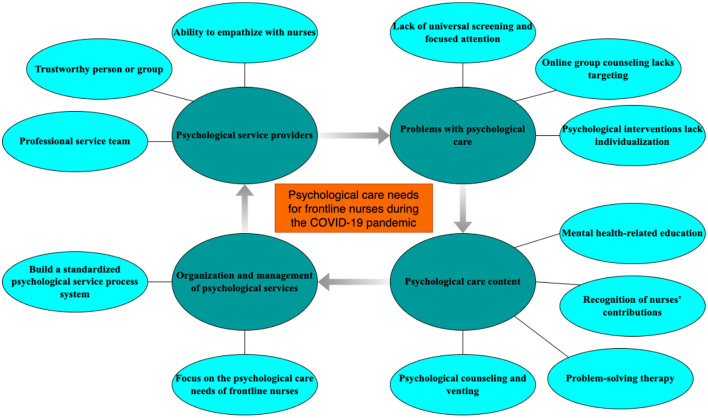
Themes and categories of psychological care needs for frontline nurses during the COVID-19 pandemic.

A total of 15 nurses participated in interviews, including 3 males and 12 females, with an average age of 33 years and an average working experience of 11.4 years were recruited, and sociodemographic characteristics are shown in Table 1. In this study, the analyses revealed 184 codes and four themes: psychological service providers, problems with psychological care, psychological care content, organization and management of psychological services. The themes and categories are shown in Figure 1.

**Table 1:** Participants' characteristics (*n* = 15).

**Characteristics**	Means (SD)/F (%)*
Age (years)	33 (5.03)
**Gender**
Male	3 (20.0)
Female	12 (80.0)
**Marriage**
Married	11 (73.3)
Unmarried	4 (26.7)
**Highest educational level**
Bachelor's degree	11 (73.3)
Master's degree	4 (26.7)
**Professional title**
Nurse practitioner	6 (40.0)
Nurse in charge	9 (60.0)
**Work experience (years)**
2–5	2 (13.3)
6–10	5 (33.3)
11–15	6 (40.0)
More than 15	2 (13.3)
**Specialty**
Chronic care	4 (26.7)
Surgical care	5 (33.3)
Intensive care	3 (20.0)
Outpatient and emergency	3 (20.0)

* SD, standard deviation; F, frequency.

### Psychological service providers

#### Professional service team

Some participants were of the opinion that the psychological care services they received were not professional enough and they hoped that the professional team would provide them with professional psychological care. At the same time, participants who received psychological care provided by the mental health nurses of our hospital and services provided by psychologists were of the opinion that there was a difference in care and were eager to receive more professional care services. They also opined that nurses with more serious psychological problems need more professional care services from psychologists. Moreover, nurses felt at ease if there were professionals with expertise on pandemic prevention and control in the psychological care team.

“*I think that the professional guidance they (mental health nurse) give is not professional enough, at least it can't achieve the desired effect so that people can psychologically relax… It is necessary to have the relevant professional experience because I find that services provided by professional and non-professional individuals are very different” (Interviewee 12, Age:27, Male)*

“*Maybe, it would be better for external professional staff to provide guidance… However, the hospital psychological care team should include some psychological experts, and for the teams aimed at controlling this pandemic, there must be at least two experts on epidemic prevention and control” (Interviewee 15, Age:39, Female)*

#### Trustworthy person or group

Most participants feel that the prerequisite for their willingness to actively seek psychological help is trust. If the psychological services are provided by a stranger, they may not freely express their inner feelings or even seek psychological help. They highly regard confidentiality and are, therefore, less trusting, especially to strangers. If they are not sure of confidentiality from the service provider, they would rather not seek, or seek the service from a third-party organization without interest.

“*Two people can freely talk with each other because they have a certain foundation, that is, there is a foundation of trust…… For example, if you are a professional psychologist, but I'm not familiar with you, I might not be open to discussing exactly why I am seeking your help”(Interviewee 10, Age:30, Female)*

“*However, to a third-party service provider, I can confide in them, I can talk about all kinds of bad things and troubles encountered in all kinds of work. I can be open to them, and I don't have to worry about them telling my leader, because they only provide psychological counseling to me. They are of a charitable nature without any conflict of interest with the hospital, therefore, I don't need to worry” (Interviewee 12, Age:27, Male)*

#### Ability to empathize with nurses

Most participants are inclined to speaking to people who can understand them, especially familiar colleagues who have the same experiences. This kind of communication lets them know that they are not alone, and that the problems they face are widespread. They especially think that colleagues with rich mental and psychological experiences can keenly capture their ideas, understand them, empathize with them and instill hope in them.

“*If they (psychological service providers) don't understand the medical industry, it won't work” (Interviewee 7, Age:41, Female)*

“*I am of the opinion that communication between colleagues is very important, since they have many things in common, they can easily understand each other” (Interviewee 15, Age:39, Female)*

### Problems with psychological care

#### Lack of universal screening and focused attention

Due to the COVID-19 pandemic, the high number of patients and shortage of medical staff, the current psychological care is limited to nurses who take the initiative to report and seek professional consulting. All nurses are not screened. Some participants felt that due to various reasons, some of them would be unwilling to actively seek help. Active reporting may not guarantee that all nurses in need will get help. At the same time, they felt that it was understandable that there was no universal screening. After all, there were a lot of infected people at the time, and the medical staff were in short supply. Many medical staff were also infected with COVID-19, or were isolated as close contacts, and there were no extra mental health nurses with a speciality in providing psychological services.

“*If there is a need, you can individually contact them… However, a person who is depressed, or with psychological problems may be unwilling to inform others about their condition” (Interviewee 4, Age:29, Female)*

“*Due to the pandemic, everyone is very busy and there is a strain on human resources, therefore, there is no general screening” (Interviewee 5, Age:37, Female)*

The nurses were of the opinion that if it is impossible to achieve universal screening, we should focus on departments and populations with higher exposure risks, so as to be able to detect problematic nurses early and intervene while others felt that even if it was necessary or not, all nurses should be screened and given psychological counseling to prevent possible psychological problems.

“*We don't have to wait until the problem arises for treatment. We may try and do an earlier diagnosis to find out if the problem exists, and thereby, intervene” (Interviewee 14, Age:38, Female)*

“*The most predisposed nurses, for example, are the ones in the fever clinic, which also has an isolation ward. This kind of place should be equipped with a special psychological team… After each shift, the nurses should be provided with psychological care services, whether necessary or not” (Interviewee 12, Age:27, Male)*

#### Online group counseling lacks targeting

Due to the highly contagious nature of COVID-19, we found that the current psychological assistance for frontline nurses is through online consultation. WeChat group counseling is currently the most commonly used form of psychological counseling. Some nurses believe that the use of WeChat groups for group counseling has limited outcomes, because group counseling does not address the needs of each individual nurse. At the same time, since everyone's working hours are different, it is difficult to communicate in depth, and it is impossible to get timely and true feedback from everyone. Sometimes, WeChat group counseling makes people feel useless and annoying. First-line nurses need targeted psychological counseling and help, non-mechanical one-to-one commu-nication with psychological support through text messages enhances recovery.

“*In WeChat group counseling, there is no way in which targeted counseling can be conducted…I think the most important thing at this time is psychological relaxation and decompression, however, by looking at what is posted in the WeChat group, not only is there no decompression, it is also useless and annoying” (Interviewee 12, Age:27, Male)*

#### Psychological interventions lack individualization

Most nurses believe that graded interventions should be performed depending on the evaluation results of clinical nurses' mental status, and that the frequency and duration of interventions should also be determined according to the mental status of nurses and the effect of each intervention.

“*When stress occur, I feel very anxious. I feel that at the initial stress stage, interventions are frequently needed, and that they will be conducted depending on the degree of psychological stress. For example, psychological stress is how many points a nurse has scored, and if the evaluation is serious, he/she can see a psychologist every day. Sometimes, people have a stronger psychological tolerance, and then they can slightly reduce their frequency of seeing a psychologist” (Interviewee 1, Age:27, Female)*

### Psychological care content

#### Mental health-related education

The vast majority of participants mentioned that they needed mental health-related education, with some practical skills for relieving stress and relaxation. Some nurses actively sought mental health-related knowledge, or studied the knowledge provided by the hospital. In addition to improving their own mental health, nurses were also eager to gain mental health-related knowledge in order to help others.

“*I think when I was struggling with anxiety, I really needed someone to support me, someone to tell me how to deal with it. When you are anxious, when you cannot sleep at night… how should you resolve this aspect, where should your focus be? There is a need to learn some methods of deep breathing, or some kind of righteous thoughts, or other various methods that can enable you relax and focus on your anxiety” (Interviewee 14, Age:38, Female)*

“*I had saw the psychological health propaganda video sent by the Health Commission of Hubei Province, and it did a good job. I think everyone should be suggested to have a look at it” (Interviewee 10, Age:30, Female)*

Some participants believed that the cause of psychological problems may be due to a lack of mental health-related education. Nurses with the mental health-related education are not prone to stigma. Therefore, some participants suggested that general mental health-related education should be administered to alleviate nurses' stigma and prejudice. In addition to mental health-related education, they also believe that the relevant psychological knowledge can also guide them psychologically.

“*I think that Chinese people's* mental health-related *education is not enough. When people seek psychological help, they will think that I am mentally ill” (Interviewee 11, Age:37, Male)*

“*If you know the knowledge about disease prevention and control, it will help you maintain a good mental state” (Interviewee 5, Age:37, Female)*

#### Recognition of nurses' contributions

Most participants need others to appreciate their contributions, thereby, validating their efforts, especially if the appreciation is from leaders and people with great influence. Patients, patient's families, and social recognition can also improve nurses' psychological conditions.

“*Leaders only need to say one sentence, sometimes one sentence is very helpful to everyone, right? Just one sentence of leadership: “you have worked hard, you are really tired, go back and rest early”. You will feel better” (Interviewee 2, Age:32, Male)*

“*It means that every time you care for a patient with a novel coronavirus, your own dedication will be recognized by leadership and understood by patients, families and colleagues” (Interviewee 8, Age:24, Female)*

#### Problem-solving therapy

Some participants were of the opinion that problem-solving was easier for relieving their psychological pressures. When a problem is resolved or eased, they feel relieved. They even think that simple psychological counseling is not very effective without solving actual problems, because, if the root cause of psychological distress is not solved, then, psychological problems will easily recur. Moreover, participants understood that some problems could not be solved at the exact time they occur, but they thought that even if the problem was unsolvable, there was a need to have a clear communication to let them know that everyone was working hard to solve it.

“*For example, we experienced a lot of difficulties at that time. When we were in the west campus of the hospital, there was no shuttle bus. Then they communicated with the leaders of the west campus and changed the time of the shuttle bus. Our colleagues could go home and have a good sleep after the night shift at night…… Then it made me feel very, very relieved” (Interviewee 1, Age:27, Female)*

“*Let me give you an example, the negotiator that talk to those who want to jump off buildings, and are able to channel them psychologically, and then save them. But, in the long-run, it may still be necessary for some people around them to pull them out of that predicament. I think that if the problem in their own personal work is not solved, the psychological problem will recur” (Interviewee 4, Age:29, Female)*

#### Psychological counseling and venting

Some participants were of the opinion that psychological consultation and catharsis can alleviate negative emotions. The purpose of this kind of communication is not necessarily to solve problems. Verbal comfort can alleviate many negative emotions. Some nurses simply want to vent. They also feel that by collecting their thoughts and opinions, the hospital is paying attention to them, thereby, giving them an official outlet.

“*Sometimes I communicate with colleagues and leaders who are senior people in psychology, and then sometimes I talk to them in the way of chatting, so as to relieve an anxiety or a heart negative emotions” (Interviewee 10, Age:30, Female)*

“*At that time, during the COVID-19 pandemic, there was no way to convey many of our voices and no channel to vent them. Later, some people confided in the WeChat work group. Including I may also confide in venting a little, after venting and then went home is fine. Maybe some people just need a channel of venting” (Interviewee 7, Age:41, Female)*

### Organization and management of psychological services

#### Focus on the psychological care needs of frontline nurses

Participants believed that psychological care was very important during the pandemic. However, they felt that the hospital did not pay enough attention to the provision of psychological services, especially psychological care for frontline nurses. With the emergence of psychological problems, a series of countermeasures were developed. They hope that the hospital will pay more attention to the psychological needs of frontline nurses.

“*I think psychological counseling teams should be organized during the epidemic to carry out psychological counseling activities in the Fang Cabin, but I have not come across them and I don't know how to get in touch with them. In fact, from the Wenchuan earthquake in 2008 to now, this kind of psychological counseling groups to help frontline disaster aid workers is very small” (Interviewee 11, Age:37, Male)*

“*In the latter stages, several aspects, such as psychological counseling calls, slowly started to be established. Since many people were psychologically affected, these countermeasures were slowly started” (Interviewee 14, Age:38, Female)*

#### Build a standardized psychological service process system

Due to a lack of the relevant hospital experience and emergency plans associated with psychological care, the hospital's management of nurses' psychological care in the course of the COVID-19 pandemic is rather chaotic. Some nurses hope for a standardized process system that allows them to clarify their roles and know who to seek help from, and how to seek help. They also hope that the processing system for psychological services is sufficiently complete and operable to cover not only the outbreak of the pandemic and the prevention before the outbreak, but also the recovery after the outbreak.

“*Before going to the front line, we need to be provided with some psychological counseling to help us better adapt to the front line work. We also need to be provided with psychological support during the course of our work, or when at home for isolation. I think there needs to be a standardized process system” (Interviewee 5, Age:37, Female)*

“*It did not have any emergency plans in advance, and it did not consider this situation…there is a more practical psychological service process in case of emergencies, it would be better. Just like the suicide of a patient, if a patient has a suicidal tendency, we immediately start the suicide process of the patient, and we will clearly know what to do and how to do it” (Interviewee 7, Age:41, Female)*

## Discussion

To our knowledge, this is the first study that explores the psychological care needs of frontline nurses during the COVID-19 pandemic in China. These findings provide a holistic view of psychological services for frontline nurses during the COVID-19 pandemic.

The first step in emergency support is to find out who is the right person for rescue operations (24). Our study found that some nurses wanted more professional people to provide psychological services to them, and this finding is consistent with the results of a questionnaire survey conducted by Kang et al. (10) among healthcare workers in Wuhan. The reason may be that people with stronger professional skills can provide more effective psychological services and more trusted by the public. Some participants felt that counseling provided by mental health nurses had limited usefulness. This could be attributed to the fact that there is a certain gap between mental health nurses and professional psychological service providers in terms of professional ability (30). Similarly, in the guidelines for emergency psychological crisis intervention in pneumonia pandemic with novel coronavirus infection issued by the Chinese centers for disease control and prevention (CDC), it is emphasized that professional psychologists should be organized to intervene with healthcare workers who show psychological symptoms (31). Mental health nurses are composed of nurses with counselor certificates from various departments of the hospital. They set up psychological care team under the leadership of the hospital nursing department to provide psychological counseling services to clinical nurses with psychological problems in general, but they could not meet the demand for frontline nurses' psychological services needs during the COVID-19 pandemic.

In addition to professional skills, first-line nurses believe that people they trust and those who can empathize with them can provide psychological support. This finding is consistent with that of Setareh Forouzan et al. and Powell and Clarke on ordinary people receiving mental health services (32, 33). The reason could be that it is easier to open up to people you trust and who can understand you, and have deeper communication as well as exchanges that are conducive for the venting of bad emotions and the search for suitable relief methods. Therefore, in addition to considering mental health nurses' professional skills when forming a psychological service team in a hospital, the empathy of nurses and whether they are able to be trusted should also be considered.

Assessment screening is the central theme of psychological nursing practice and the basis for interventions (34). However, due to the COVID-19 pandemic, clinical workload is heavy, and psychological service providers are strained. It may not be practical to conduct a comprehensive psychological screening of frontline nurses, so a self-reported approach to psychological services was used during the COVID-19. However, self-report measures limit the reach of psychological services, since some nurses may have a stigma and not actively seek psychological help (10). This may lead to a more serious mental illness that exerts a serious burden on the health system. Therefore, we recommend the development of a brief and rapid psychological screening tool for nurses to achieve the purpose of general psychological screening in emergency situations. In addition, we suggest that screening should be done in key departments and populations with relatively high exposure levels, and depending on the screening results, first-line nurses should be given priority interventions.

Online psychological counseling can help relieve acute stress. In a survey conducted by Cai et al. (35), more than three-fifths of nurses believed that online psychological counseling had an inhibitory effect on anxiety, insomnia, and PTSD symptoms. Zeng et al. (36) proposed that some medical staff can benefit from online consultations and that supportive WeChat groups with psychologists should be established for sharing and communication. Due to the highly contagious nature of COVID-19, and the special work of medical staff, online consultation is considered a better way for the provision of mental health services. However, this study revealed that online communication methods such WeChat group counseling has a limited effect. All participants did not receive one-on-one counseling via video, they were of the opinion that the WeChat group counseling method cannot target an individual, and that everyone's arbitrary speech in the group may lead to negative emotions. They prefer targeted individual consultations, consistent with the findings of Setareh Forouzan et al. (32) and Kang et al. (10). This is because there are some situations where nurses are unwilling to express themselves in the group, and therefore, personal online consultation via video can be a better option.

Nurses need mental health knowledge to care for and help others (22). By being given adequate information and sufficiently trained, the competency of nurses can be improved (1). We found that knowledge education required by nurses includes psychological and professional knowledge. Most of the frontline nurses had not received any mental health training (37). In terms of psychological knowledge, most nurses just wanted to use it to help others and themselves. This is consistent with the results of a previous survey (10). This could be due to a sense of responsibility and the mission of the nursing profession. In addition to helping themselves, they also look forward to serving patients and those around them. Furthermore, they believe that providing the relevant professional knowledge information is also helpful for their psychological protection. A recent meta-analysis confirmed this finding (38). This is possibly because the relevant professional knowledge information will reduce their sense of uncertainty and increase their confidence in fighting the disease. In the guidelines for emergency psychological crisis intervention in pneumonia pandemic with novel coronavirus infection issued by the Chinese CDC, it is also highlighted that psychological crisis intervention training is conducted before health care workers participate in the rescue, to understand the stress response and learn methods to cope with stress and regulate emotions (31). Therefore, we suggest that hospitals should routinely conduct regular psychological-related knowledge training to educate clinical nurses about psychological knowledge, especially after major public health emergencies, and provide emergency training for nurses in psychological crisis to better maintain nurses' mental health.

Problem-solving therapy is a brief evidence- and strength-based psychotherapy that has received increasing support for its effectiveness in managing depression and anxiety among primary care (39). We found that nurses have a strong preference for problem-solving therapy. They expect hospital administrators to address the difficulties they face in their work (such as supplies, shuttles, accommodations, etc.), which will make them more focused on their work itself, resulting in less stress and anxiety.

Psychological counseling and venting are convenient and effective methods. Mo et al. (40) recommended that nurses should encourage each other, discuss and share their feelings as well as experiences with someone on time, and vent negative emotions. Chan and Huak (41) indicated that being able to give management feedback can also help vent negative emotions. Our study also found that nurses wanted to have a channel to communicate their opinions to hospital administrators. We suggest that hospitals should establish channels to collect nurses' demands, pay more attention to the psychological needs of front-line nurses, and encourage them to speak their minds and vent their bad emotions.

In most countries, mental health services receive less attention than physical health (42). Previously, few interventions were put in place to meet the mental health needs of medical staff in areas affected by Ebola virus infection (43), and we also obtained similar finding. The reason may be that serious consequences such as death and disability caused by physical diseases are more obvious, while the psychological diseases are more hidden. Hospitals should pay more attention to the mental health of frontline nurses, understand the adverse effects of nurses' mental state on the healthcare system, and take active measures to adjust the mental state of frontline nurses to better control emergencies. Management of public health emergencies should adopt a systematic approach (44). However, there is currently no standardized operating procedure for Chinese hospitals to respond to emergency psychological service systems for public health emergencies. Once a public health emergency occurs, nurses do not know what to do first and whom to ask for help from when they have psychological problems. Therefore, we recommend that hospitals should establish complete and standardized emergency psychological service systems for public health emergencies. This system includes not only the response to public health emergencies, but also prevention of emergencies, preparations for when they come, and recovery after they have happened, so as to provide frontline nurses with comprehensive and individualized psychological services that can better respond to public health emergencies.

### Limitations

Inevitably, this study had limitations, three of which are noteworthy. First, we focused on the psychological care needs of frontline nurses during the COVID-19 pandemic. More studies should be done to elucidate on the psychological care needs before and after the COVID-19 epidemic. Second, this study only included the views of nurses who received psychological care services during the pandemic. Nurses who did not receive psychological care services during the pandemic also had their own unique psychological needs. Future studies should involve this population. Finally, this study was based on nurses of one hospital in Wuhan. However, China's regional and social environments are relatively complex, and the hospitals of various sizes and levels have important differences. Therefore, the applicability of the study's conclusions needs further verification. All in all, a larger range of qualitative and quantitative studies will need to be conducted in the future to extend the credibility of our existing analyses.

## Conclusion

The current study provides a comprehensive understanding of the psychological care needs for clinical frontline nurses during the COVID-19 pandemic. Across these conceptual themes, we learned that frontline nurses need professional psychological service providers to provide them with psychological counseling services, and need psychological support from trustworthy people and someone who can empathize with them. They want targeted and individualized psychological interventions in the form of universal screening, based on the individual psychological state of the nurse. At the same time, they desire mental health-related education to help themselves and others, they need to be recognized by others, and they want the hospital to be able to address some of the practical issues that are troubling them psychologically, and to be able to talk about their psychological problems rather than be criticized when they arise. Likewise, they want their psychological problems to be taken seriously by hospital leaders and a clear and standardized psychological service system to let them know what to do in case of psychological problems. By doing this, frontline nurses would be better equipped to face ongoing psychological crisis as COVID-19 continues.

## Data availability statement

The original contributions presented in the study are included in the article/Supplementary material, further inquiries can be directed to the corresponding author.

## Ethics statement

The studies involving human participants were reviewed and approved by Institutional Review Board of Union Hospital, Tongji Medical College, Huazhong University of Science and Technology (IRB number: 2021S003). The patients/participants provided their written informed consent to participate in this study. Written informed consent was obtained from the individual (s) for the publication of any potentially identifiable images or data included in this article.

## Author contributions

CD and LW: conceptualization, data collection, and transcribing. ZG: methodology. CD: software, formal analysis, writing—review, and editing. LW, ZG, and YC: validation. YC: resources and data curation. LW: writing—original draft preparation. JJ: supervision and project administration. All authors have read and agreed to the published version of the manuscript. All authors contributed to the article and approved the submitted version.

## Funding

This work was supported by Health Commission of Hubei Province Scientific Research Project (WJ2021M241).

## Conflict of interest

The authors declare that the research was conducted in the absence of any commercial or financial relationships that could be construed as a potential conflict of interest.

## Publisher's note

All claims expressed in this article are solely those of the authors and do not necessarily represent those of their affiliated organizations, or those of the publisher, the editors and the reviewers. Any product that may be evaluated in this article, or claim that may be made by its manufacturer, is not guaranteed or endorsed by the publisher.
