# Supplementary material for: Psychological care needs for frontline nurses during the COVID-19 pandemic: A qualitative study
Source: Front Public Health. 2022 Nov 10;10:1043515. doi: 10.3389/fpubh.2022.1043515 (PMC9686294; doi:10.3389/fpubh.2022.1043515)
Supplement: Supplementary file 1 [file Table_1.DOCX]

Supplementary Material

# Supplementary File 1: Consolidated criteria for Reporting Qualitative research (COREQ) : 32-item checklist

| No. | Item | Guide Questions/Description | Reported. |
| --- | --- | --- | --- |
| Domain 1: Research team and reflexivity  Personal characteristics | | | |
| 1 | Interviewer/facilitator | Which author/s conducted the interview or focus group? | Chuanqi Ding and Limin Wang |
| 2 | Credentials | What were the researcher’s credentials? E.g. PhD, MD | Nursing master |
| 3 | Occupation | What was their occupation at the time of the study? | Nurse. |
| 4 | Gender | Was the researcher male or female? | 4Female, 1Male |
| 5 | Experience and training | What experience or training did the researcher have? | The first authors had special training in interviewing and all the others have extensive interview and qualitative research experience. |
| Relationship with participants | | | |
| 6 | Relationship established | Was a relationship established prior to study commencement? | Yes. |
| 7 | Participant knowledge of the interviewer | What did the participants know about the researcher? e.g. personal goals, reasons for doing the research | Purpose and content of the study |
| 8 | Interviewer characteristics | What characteristics were reported about the interviewer/facilitator? e.g. Bias, assumptions, reasons and interests in the research topic | Yes. |
| Domain 2: Study design  Theoretical framework | | | |
| 9 | Methodological orientation and Theory | What methodological orientation was stated to underpin the study? e.g. grounded theory, discourse analysis, ethnography, phenomenology, content analysis | Qualitative descriptive study and conventional content analysis |
| Participant selection | | | |
| 10 | Sampling | How were participants selected? e.g. purposive, convenience, consecutive, snowball | Purposive. |
| 11 | Method of approach | How were participants approached? e.g. face-to-face, telephone, mail, email | Face-to-face and telephone |
| 12 | Sample size | How many participants were in the study? | 15 participants. |
| 13 | Non-participation | How many people refused to participate or dropped out? Reasons? | No participant refused to participate or dropped out. |
| Setting | | | |
| 14 | Setting of data collection | Where was the data collected? e.g. home, clinic, workplace | Workplace and home |
| 15 | Presence of nonparticipants | Was anyone else present besides the participants and researchers? | No |
| 16 | Description of sample | What are the important characteristics of the sample? e.g. demographic data, date | Described in Table 1 |
| Data collection | | | |
| 17 | Interview guide | Were questions, prompts, guides provided by the authors? Was it pilot tested? | Yes. |
| 18 | Repeat interviews | Were repeat inter views carried out? If yes, how many? | No. |
| 19 | Audio/visual recording | Did the research use audio or visual recording to collect the data? | Yes. |
| 20 | Field notes | Were field notes made during and/or after the interview or focus group? | Yes. |
| 21 | Duration | What was the duration of the inter views or focus group? | 40–60minutes. |
| 22 | Data saturation | Was data saturation discussed? | Yes. |
| 23 | Transcripts returned | Were transcripts returned to participants for comment and/or correction? | No. |
| Domain 3: analysis and findings  Data analysis | | | |
| 24 | Number of data coders | How many data coders coded the data? | Two authors. |
| 25 | Description of the coding tree | Did authors provide a description of the coding tree? | N/A |
| 26 | Derivation of themes | Were themes identified in advance or derived from the data? | Yes |
| 27 | Software | What software, if applicable, was used to manage the data? | Nvivo 12 (QSR) |
| 28 | Participant checking | Did participants provide feedback on the findings? | No. |
| Reporting | |  |  |
| 29 | Quotations presented | Were participant quotations presented to illustrate the themes/findings? Was each quotation identified? e.g. participant number | Yes. |
| 30 | Data and findings consistent | Was there consistency between the data presented and the findings? | Yes. |
| 31 | Clarity of major themes | Were major themes clearly presented in the findings? | Yes. |
| 32 | Clarity of minor themes | Is there a description of diverse cases or discussion of minor themes? | Yes. |

Developed from: Tong, A., Sainsbury, P., & Craig, J. (2007). Consolidated criteria for reporting qualitative research (COREQ): a 32-item checklist for interviews and focus groups. International Journal for Quality in Health Care, 19(6), 349-357. doi: 10.1093/intqhc/mzm042
